# High Resolution ^31^P NMR Spectroscopy Generates a Quantitative Evolution Profile of Phosphorous Translocation in Germinating Sesame Seed

**DOI:** 10.1038/s41598-017-18722-y

**Published:** 2018-01-10

**Authors:** Honghao Cai, Wei-Gang Chuang, Xiaohong Cui, Ren-Hao Cheng, Kuohsun Chiu, Zhong Chen, Shangwu Ding

**Affiliations:** 1 Department of Chemistry and Center for Nanoscience and Nanotechnology, National Sun Yat-sen University, 70 Lien-Hai Road, Kaohsiung, Taiwan 80424 Republic of China; 2 School of Science, Jimei University, 183 Yinjiang Road, Xiamen, China; 3 Department of Electronic Science, Fujian Provincial Key Laboratory of Plasma and Magnetic Resonance, State Key Laboratory of Physical Chemistry of Solid Surfaces, Xiamen University, Xiamen, China; 4 Department and Graduate Institute of Aquaculture, National Kaohsiung Marine University, Kaohsiung, Taiwan 811, Republic of China

## Abstract

Phosphorus metabolism and circulation are essential bio-physicochemical processes during development of a plant and have been extensively studied and known to be affected by temperature, humidity, lighting, hormones etc. However, a quantitative description of how various phosphorous species evolve over time has not been reported. In this work, a combined ^31^P liquid and solid state NMR spectroscopic methodology is employed, supported by a new extraction scheme and data analysis method, to carry out a quantitative investigation of phosphorous circulation in germinating sesame seeds in dark and under illumination with and without adding a growth hormone. The spectra show that only slight changes occur for phosphorous metabolism at the initial stage but a rapid change takes place between 48–96 hours after germination is started. The metabolism is found to be temperature dependent and affected by illumination and hormone. However, neither illumination nor hormone affects the final residual concentration of phytin. Moreover, phytin does not flow out of cotyledon and the phosphorous flowing to other parts of the plant is always in the inorganic form. The overall evolution profile of phytate consumption is found to be a Gaussian decaying function. These findings can be explained with a dynamic model on phytin conversion.

## Introduction

Phosphorous is an essential element for organisms, involving many important physiological functions such as energy storage and transfer, cellular membrane, skeletal support etc.^1–3^. In a plant, phosphorous accounts for about 0.2–0.5% of total mass and is the most important element only next to nitrogen^3–5^. Therefore, for over one century, the research on phosphorous metabolism and translocation of phosphorous has been a vitally important subject and much advance has been achieved on phosphorous biochemistry, biophysics and physiology^1–6^.

However, there are still many open questions unanswered yet, e.g. the spatiotemporal details of a specific metabolism process and interactions between phosphorous-containing molecules/ions and other biomolecules^6–11^. Indeed, for plants, how phosphorous is taken up from soil and how it is mobilized and translocated during germination of a seed are not fully understood. These questions have fundamental significance for plant biochemistry and physiology. They are also keenly related to the yield and nutrition composition of crops, genetic modification of crops, environmental ecology, and phosphorous conservation on land etc^12–16^. A central and widely studied subject in phosphorous metabolism is related to the destiny of phytate (phytin)^6–16^.

While various experimental techniques have been used to study the biophysical chemistry of key biomolecules such as phytate, NMR spectroscopy has been shown a unique tool^17–19^, mainly because it can obtain structural and dynamics information at the atomic level simultaneously. When combined with prudential isotope labelling, solid state NMR spectroscopy has been proven a powerful method for studying the biochemistry of plants *in vitro* or *in vivo*, as beautifully illustrated by Schaefer group^20–22^. In the past years, NMR spectroscopy has been employed to investigate the phosphorous fate and phytate dynamics in a variety of systems from soil, manure, poultry litter, foods and fruits to living organisms^23–28^. Shand *et al*.^17^ applied ^31^P solid state NMR to study phosphorous distribution in different types of soils. Cade-Menun *et al*.^18,19^ investigated the destiny of phosphorous in soil and its affection on environment and agriculture. Hunger *et al*.^20^ analyzed the phosphorous in poultry litter using solid state NMR spectroscopy. Jayasundera *et al*.^21^ and He *et al*.^22^ measured different forms of phosphorous in dairy manures.

He *et al*.^23^ studied the structural characteristics of a series of metal species of phytate using solid state NMR and x-ray absorption near edge structure (XANES). They showed that intensive sidebands in solid state NMR spectra, indicating highly anisotropic microenvironment of metal species of phytate. The phytate degradations by lactic acid bacteria in yeast and dough fermentation were measured by Reale *et al*.^24^. The phosphorous distribution and evolution in wetland was studied by Cheesman *et al*.^25^.

More advanced ^31^P NMR methods such as heteronuclear NMR experiments involving ^31^P for studying the large-scale structure^26^ or complex biochemical reactions^27^ in biological systems were also demonstrated to be feasible and powerful. Gambhir *et al*.^28^ studied the phosphorous metabolites of the seeds of wheat, soybean and mustard using liquid state NMR spectroscopy. The cytoplasmic ^31^P signals over a range of moisture contents were recorded and examined. This enabled them to measure the metabolically important cytoplasmic pH value to provide clear evidence for the existence of a hypoxic state in developing seeds.

However, to the best of our knowledge, although ^31^P NMR research of living plants has been conducted in previous works^29–34^, monitoring the circulation of phosphorus over an entire course of the development of a plant seed has not been conducted. The ^31^P NMR spectroscopic results has not been analyzed in perspective of the biophysical mechanism of phosphorous metabolism and circulation. In addition, the resolution of the previous studies was not optimized which limited the quantitativeness of the spectra. In this work, therefore, we explore the application of ^31^P NMR spectroscopy to monitor the germination of sesame seed. To achieve high resolution in the NMR spectra, possible paramagnetic species were carefully removed. It is worth mentioning that quantitativeness has been a challenge in applying ^31^P NMR spectroscopy for either *in vivo* or *in vitro* research because of the diverse microenvironments for phosphorous in an organism. Insufficient quantitativeness hinders quality of research and causes misleading conclusions. In present work, quantitativeness is achieved via a novel sample preparation scheme and a modified method for data analysis, in addition to high spectral resolution. The high quality data enable us to obtain quantitative time evolution of phosphorous metabolism during the entire course of seed germination. The evolution curves can be rationalized by a practical model on the enzyme dynamics during seed germination.

## Results and Discussion

Sesame seed (*Sesamum indicum L*.) was purchased from a local vendor. Gibberellin GA_3_ (gibberellic acid, C_19_H_22_O_6_) of purity >99% was purchased from Aldrich and used without further purification. Ethylenediaminetetraacetic acid (EDTA, C_10_H_16_N_2_O_8_) for removing metal elements from the sample by chelation was purchased from Aldrich. HClO_4_ and K_2_CO_3_ used for sample processing were purchased from Aldrich. Deionized water was home made with a resistivity larger than 0.5 MΩcm.

**Figure 1:**
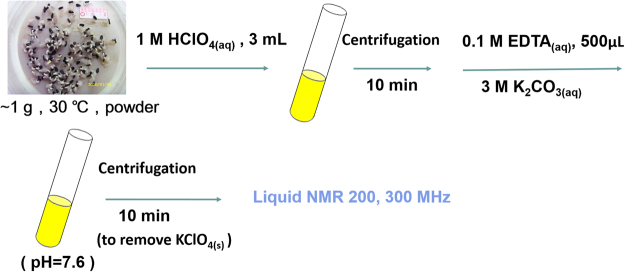
The sketchy diagram of sample preparation and experimental procedure.

The sketchy diagram of sample preparation and experimental procedure is shown in Fig. 1. About 1 g of sesame seed was taken and placed in pure water in a culture dish with controlled lighting conditions (sunlight, dark), humidity (20%) and temperature (30 °C). Timing is recorded with a computer. At the selected time point, the seed was taken out, pulverized into powder and mixed with 3 ml of 1 M HClO_4_ aqueous solution in a test tube for 10 mins. The mixture then was placed in a centrifuge (3000 rpm) for 10 mins. After centrifugation, the supernatant was taken and added with 500 μl of 0.1 M EDTA to form a homogeneous mixture so that the metal elements were chelated by EDTA. The mixture then was titrated with 3 M K_2_CO_3_ to a pH value of 7.6. The solution was again put in a centrifuge (3000 rpm) for 10 mins to separate the metal-chelating EDTA and the precipitates (e.g. of KClO_4_) from the solution. The supernatant was then used for analysis with high resolution liquid state NMR spectroscopy.

**Figure 2:**
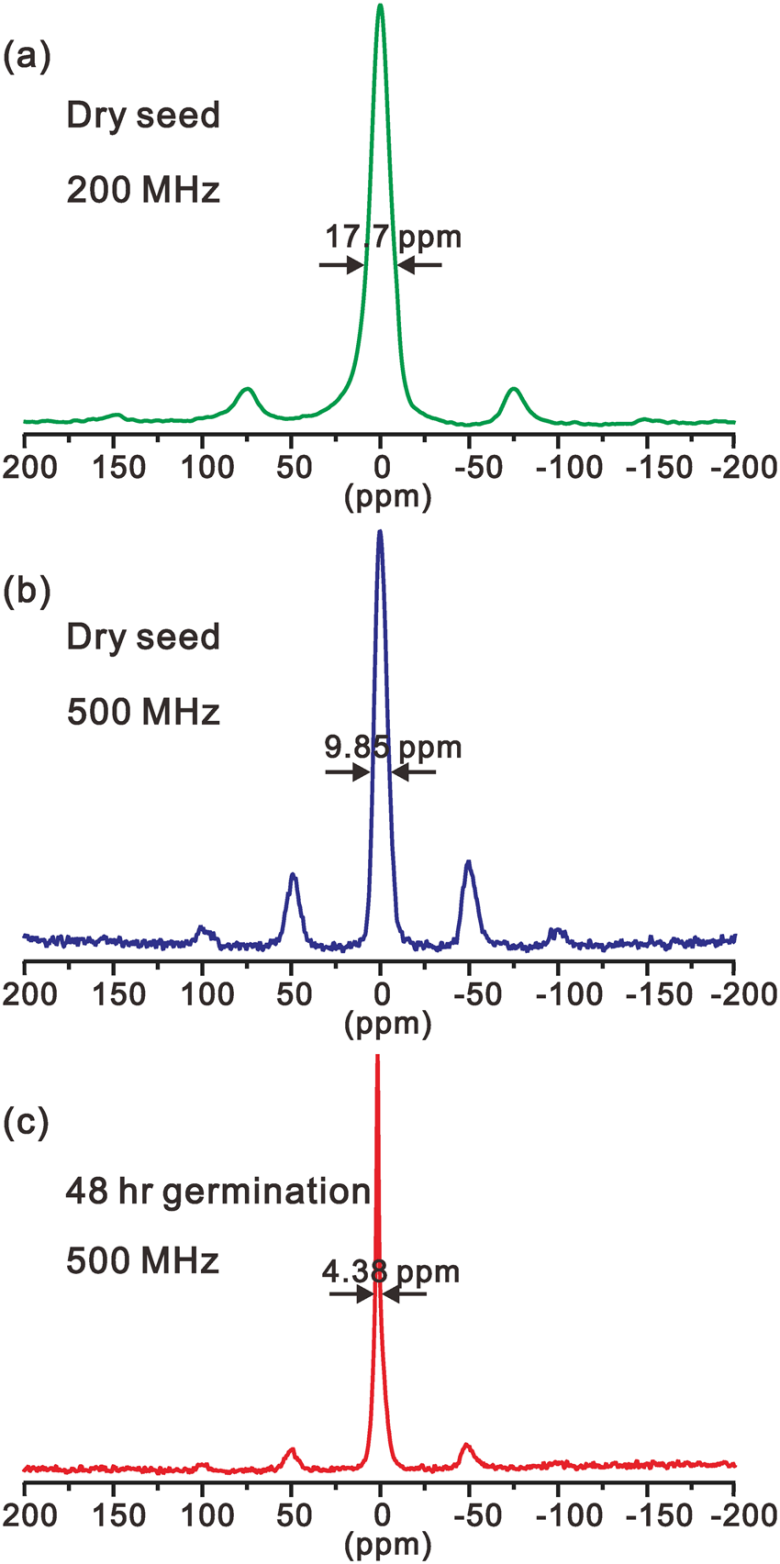
The solid state NMR spectra of dry (**a**,**b**) and germinating (**b**) sesame seed at 200 MHz (**a**) and 500 MHz (**b**,**c**). The spinning speeds were 6 kHz (**a**) and 10 kHz (**b**,**c**), respectively.

The ^31^P MAS solid state NMR spectra of sesame seed at two different magnetic fields are shown in Fig. 2. The spectra of both dry and germinating seeds show severely broadened peaks even under high speed sample spinning. There are two major origins responsible for this large broadening. The first is the strong dipolar coupling between phosphorous and paramagnetic ions such as iron and manganese, leading to paramagnetic broadening. The second is because sesame seed is highly inhomogeneous and phosphorous nuclei are located in a large number of different microenvironments, leading to a broad isotropic chemical shift dispersion. Comparing the linewidths of the central peaks of the dry seed at 200 MHz and 500 MHz, it is found that the linewidth cannot be explained with isotropic chemical shift dispersion only. With the two linewidths (17.7 ppm at 200 MHz and 9.85 ppm at 500 MHz), we can estimate that the isotropic chemical shift dispersion is 4.9 ppm and the paramagnetic broadening is about 1050 Hz. The presence of sidebands even at 10 kHz suggests large anisotropy of chemical shift of phosphorous in the seed and underscores that phosphorous spins in a sesame seed are largely immobile. However, for a germinating seed, the intensities of the sidebands are much weaker and the linewidth is much narrower, indicating that the phosphorous in a germinating seed is much more mobile than in a dry seed. The 4.38 ppm linewidth is largely from isotropic chemical shift dispersion, very close to the estimated values from the dry seeds. Although the solid state ^31^P NMR spectra provide interesting information on seed germination, the isotropic chemical shift dispersion cannot be resolved, therefore, we cannot use solid state NMR spectra to investigate the change of individual phosphorous components during germination.

### Evolution profile under illumination

**Figure 3:**
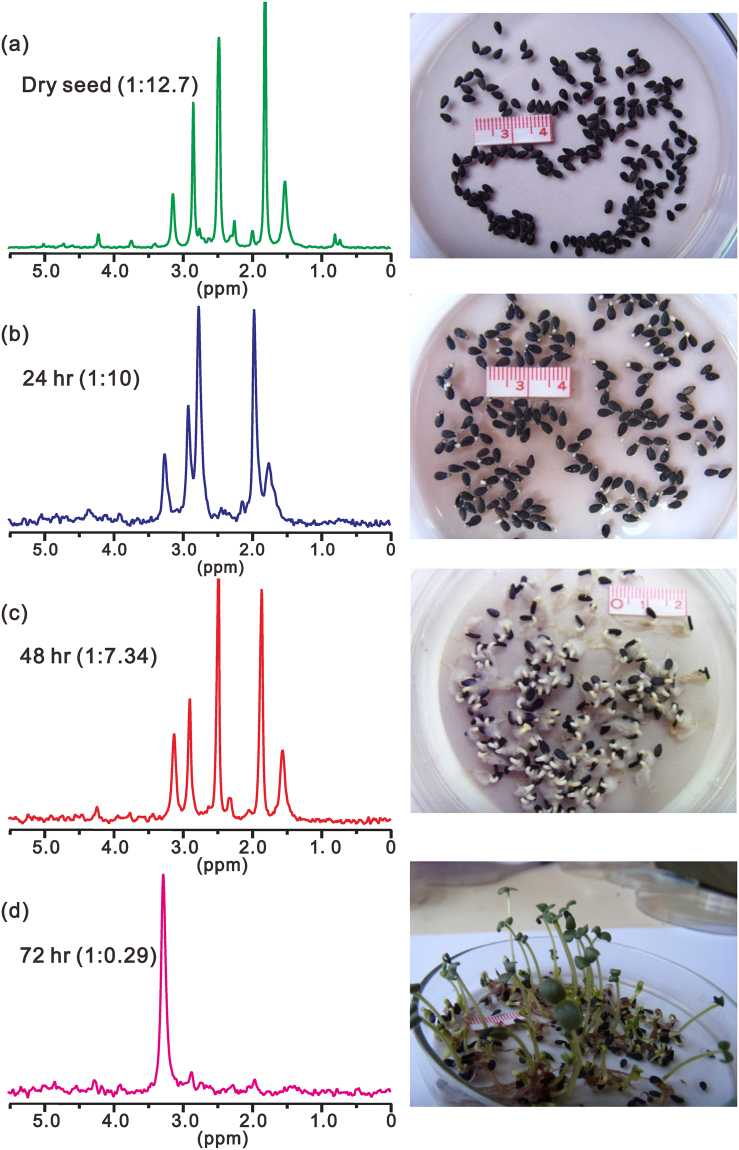
The ^31^P NMR spectra of sesame seed extracts at different times of germination in light at 0, 24, 48 and 72 hours, respectively. The right panel shows the photos of the germinating seeds taken at the corresponding time of germination.

With liquid state ^31^P NMR spectroscopy, the major components are well resolved. Shown in Fig. 3 are the ^31^P spectra of sesame seed at different germinating times under normal sunlight illumination. The peak at 3.27 ppm corresponds to the inorganic phosphorous (Pi) and the remaining peaks are from organic phosphorous Po (phytin)^24^. The intensity ratio Pi/Po at each germination time is shown in each spectrum in Fig. 3. As anticipated, with germination proceeding, this ratio increases. Initially, the increase is very slow but becomes very rapid after 48 hours of germination. After about 72 hours, virtually all phosphorous are turned into Pi and phytin is completely consumed. The overall trend of phytin degradation under illumination can be explained because illumination speeds up consumption of phytin at endosperm, particularly after the initial stage. The growing buds consume more phosphate and drives circulation of phytin from endosperm to the budding leaves.

### Evolution profile in dark

**Figure 4:**
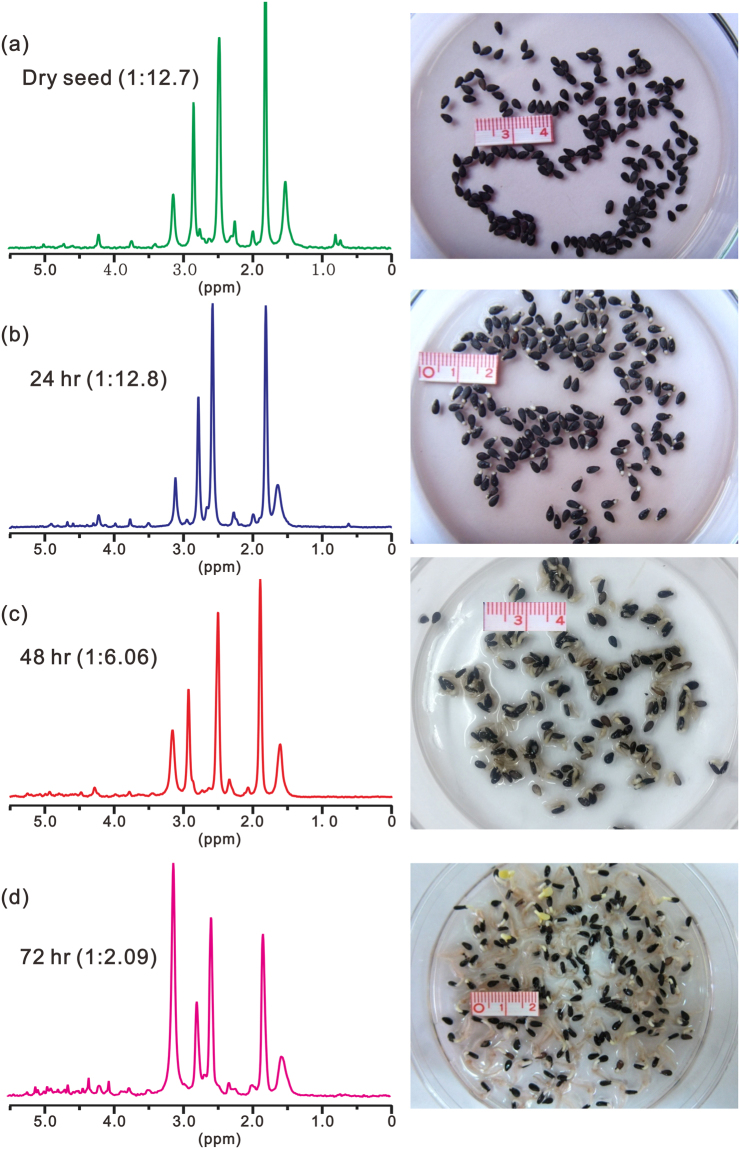
The ^31^P NMR spectra of sesame seed extracts at different times of germination in dark at 0, 24, 48 and 72 hours, respectively. The right panel shows the photos of the germinating seeds taken at the corresponding time of germination.

Figure 4 shows the ^31^P spectra of sesame seed at different germinating times in dark. Both the spectra and visual inspection (the photos) indicate that sesame seed can germinate in dark. Analogous to germination in light, with germination proceeding, Pi/Po ratio increases, initially, the increase being very slow and becoming rapid after 48 hours of germination. However, the increase of the ratio is much slower than that for seed germinating under illumination. After about 72 hours, Pi/Po is only about 1/2. It takes another 24 hours for the ratio to reach about 3 (supporting information Figure S1). Comparing Figs 3 and 4, the effect of illumination is obvious. Although sesame seed can germinate in dark, the growing speed is slower.

### Evolution profile in dark with GA_3_

**Figure 5:**
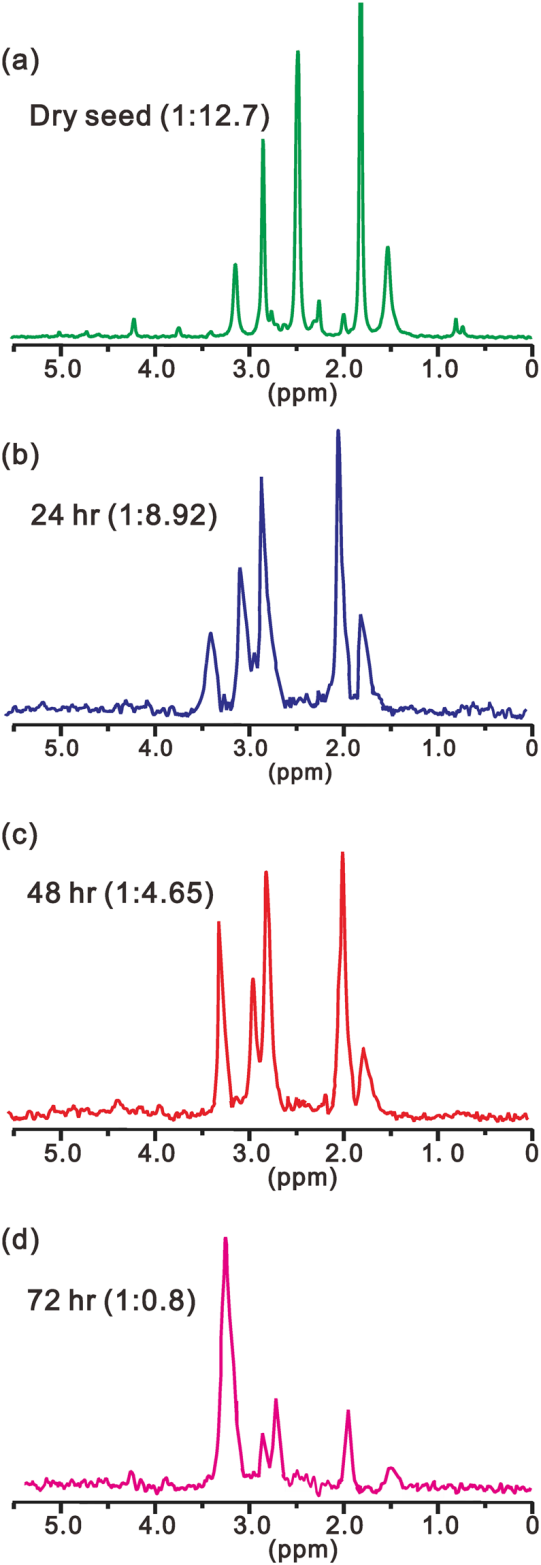
The ^31^P NMR spectra of sesame seed extracts at different times of germination in dark but with GA_3_ added in the culture liquid at 0, 24, 48 and 72 hours, respectively. The photos of the germinating seeds are very similar to those in Fig. 4.

When a sesame seed germinates in dark but the culture is added with GA_3_, the growth trend is similar to that under illumination, as shown in Fig. 5. However, some appreciable differences are worth mentioning. The initial Pi/Po ratio for germination in dark with GA_3_ is bigger than that for germination under illumination, but after about 48 hours, the former is smaller than the latter. This suggests that the effect of GA_3_ in the initial stage of germination is bigger than that of illumination, but smaller in the latter stage. This is probably because in the initial stage, GA_3_ which is imbibed into seed can affect phytase but light cannot get inside the sesame seed to affect phytase. After about 48 hours when the seed buds and can absorb light, the illumination effect is more significant than GA_3_.

From above results, it is clear that the germination speed is significantly affected by light and hormone. However, after a sufficient long time, virtually all phytin is consumed, i.e., neither illumination nor hormone affects the final residual concentration of phytin. By analyzing the spectra of seedlings (Figures S2 and S3), it is found that phytin does not flow out of cotyledon and the phosphorous flowing to other parts of the plant is always in the form of inorganic phosphorous.

### From phytin evolution profile to phytase kinetics and dynamics

**Figure 6:**
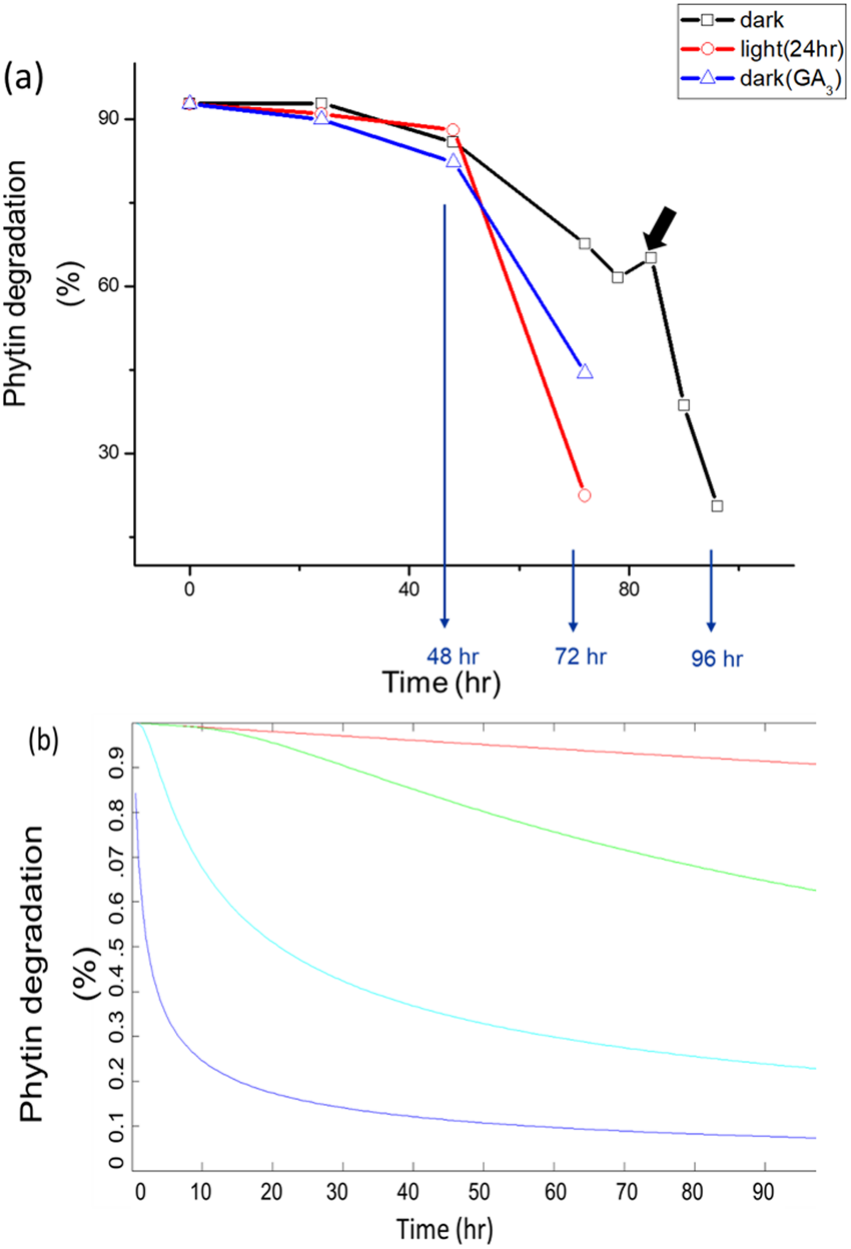
(**a**) The quantitative evolution profiles of phytin consumption in germinating seeds with different culture conditions. The obvious deviation as indicated by the thick arrow is attributed to temperature fluctuation. (**b**) Some representative results of the theoretical simulation of phytin consumption with different diffusion coefficients. The first order reaction rate was set to 10^−6^ s^−1^ and the diffusion bound was set to 20 nm. Red: without diffusion, a slow exponential decay shown; Green: with a diffusion coefficient of 10^−11^ m^2^ s^−1^; Cyan: with a diffusion coefficient of 10^−10^ m^2^ s^−1^; with a diffusion coefficient of 10^−9^ m^2^ s^−1^.

To quantitatively analyze the phosphorous evolution during germination, we plot the phytin degradation with respect to germination time for above three cases as shown in Fig. 6(A). We find that all the three curves can be fitted with a Gaussian decay function with different decaying factors. Fitting with other functions such as exponential or power law leads to much larger errors. The phytin degradation (or phytate consumption) not only quantifies the kinetics of the chemical reaction from phytin to inositol but also provides important information on the action of phytase.

**Figure 7:**
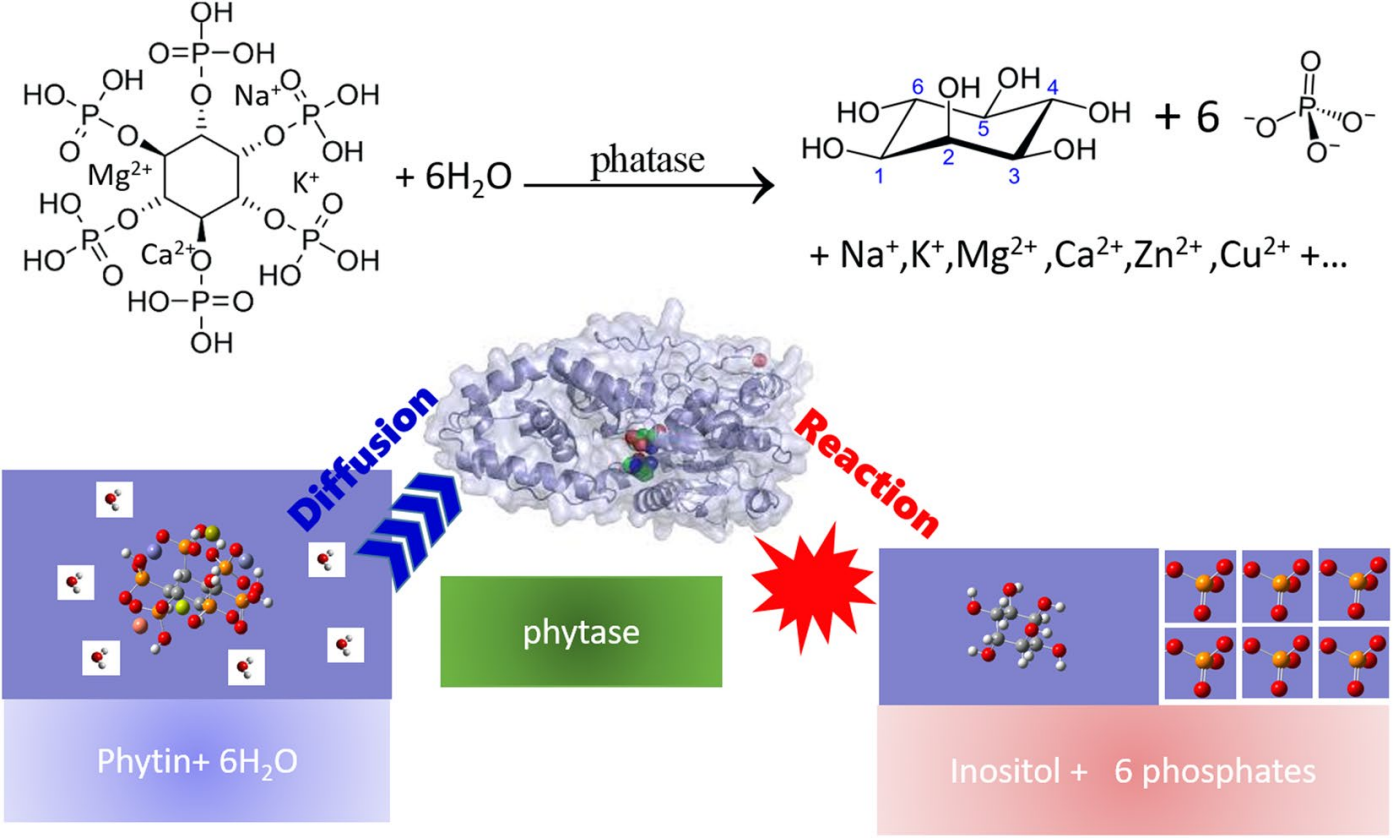
Top: The hydrolysis of phytin by the catalysis of phytase. The chelated metal cations such as Na^+^, K^+^, Mg^2+^, Ca^2+^, Fe^2+^ etc are released upon hydrolysis. Bottom: the dynamic model for explaining the consumption of phytin (or the turnout trend of inositol) as a joint action of first order kinetics from phytin to inositol and the relative diffusion of phytin with respect to phytase.

This Gaussian behavior is different from the consumption curves of fermentation of yeast or dough^24^, or in soil samples^17–19,25^. This suggests that the enzyme dynamics of phytase in sesame germination is very different from that in fermentation and non-Michaelis-Menten behavior must be taken into consideration in order to quantitatively explain our data and the data from previous studies. An obvious fact about seed germination is that the seed must be sufficiently hydrated. This provides, among other conditions, an environment that the enzymes and reactants possess high mobility. This means, during the enzyme-substrate binding, diffusion of both the substrate and enzyme must affect the overall efficiency of catalysis, hence consumption of phytin. Therefore, we use a dynamic model for phytin consumption as shown at the bottom of Fig. 7. In this model, the conversion of phytin to inositol (and other products) and the activity of phytase assume a first-order reaction-diffusion mechanism, leading to an overall Gaussian type turnout trend of inositol. We denote the concentration of inositol at any given location ***r*** and time *t* as [I](***r***, t) and the relative diffusion tensor of phytin with respect to phytase **D**, then the overall reaction-diffusion equation is expressed as

1 $$\frac{\partial [{\rm{I}}]({\bf{r}},t)}{\partial t}=\nabla {\bf{D}}\nabla [{\rm{I}}]({\bf{r}},t)-{k}_{0}[{\rm{I}}]({\bf{r}},t)$$

where *k*_0_ is the reaction rate without considering relative diffusion. There is no general analytical solution to Eq. (1) but some approximate solutions for special boundary conditions have been proposed^35,36^ and more recently some exact solutions for special cases have been reported^37^. Numerical solutions either based on solving coupled differential euqaitons^38^ or based on molecular dynamics simulation^39,40^ have also been obtained for a number of systems. We find that in our case, however, a simplified model is sufficient to provide us quantitative description of our experimental data. In this model, the diffusion tensor is assumed to be isotropic so that the problem can be converted into a one-dimensional case. For a germinating seed where the molecules have large mobility, this is an acceptable approximation. Under this assumption, the solution of Eq. (1) is given by.

2 $$[{\rm{I}}](x,t)=\frac{[{\rm{I}}](0,0){e}^{-{x}^{2}/4Dt}{e}^{-{k}_{0}t}}{A{(\pi Dt)}^{1/2}}$$

The total consumption of phytin at any given time *t* is an integration over all possible phytase that can access phytin. Suppose all phytase molecules within a fixed distance (−*b*, *b*) from phytin will promote the reaction. Then phytin consumption follows

3 $$[{\rm{I}}](t)=\frac{[{\rm{I}}](0,0){e}^{-{k}_{0}t}}{A{(\pi Dt)}^{1/2}}{\int }_{-b}^{b}{e}^{-{x}^{2}/4Dt}dx=\frac{[{\rm{I}}](0,0){e}^{-{k}_{0}t}}{A{(\pi Dt)}^{1/2}}erf(b/\sqrt{4Dt})$$

where *erf* stands for error function. Some representative solutions are plotted in Fig. 6(B) which satisfactorily reproduce the trends shown in Fig. 6(A). From Eq. (2), it is obvious that the consumption curve of phytin would follow a pure exponential trend if the relative diffusion is neglected. This result clearly shows the importance of relative diffusion between phytin and phytase in the quantitative description of phosphorous translocation in a germinating seed. It is also noteworthy that when the diffusion is too fast, as shown in the cyan and blue lines in Fig. 6(B), the Gaussian type behavior is lost and the overall trend looks more like an exponential decay.

We notice that an earlier study by Frias *et al*.^41^ on legume seeds did show non-exponential behavior, but the data are only available for three time points, making quantitative comparison infeasible.

A more recent study on legume seeds by Abdel-Gawad *et al*.^42^ shows a clear Gaussian type activity for both phytase, consistent with our above experimental results on sesame seed. Therefore, Gaussian kinetics may be a universal characteristic for phytase action for all seeds. Although studies on more seeds are required to confirm this hypothesis, the above dynamic model of phytase action seems rather reasonable.

In summary, the ^31^P solid state MAS NMR spectra show that phosphorous in a dry seed and that in a germinating seed have different microenvironments and dynamics. However, solid state spectra cannot be resolved to show different types of phosphorous. With the sample preparation protocol that provides homogeneous solutions and removes the possible paramagnetic ions, high resolution ^31^P NMR spectra of sesame seed extracts could be obtained with all major phosphorous species clearly resolved, that makes it possible to conduct quantitative analysis of phytin degradation. This quantitative study of phosphorous translocation during sesame seed germination in various conditions (in light, in dark and in GA_3_) with ^31^P NMR spectroscopy enables us to monitor the time evolution of phosphorous conversion and to obtain a quantitative evolution profile over the entire course of germination. The effects of light and GA_3_ are clearly demonstrated and compared. In all cases, the evolution profile shows clear deviation from exponential function and displays a Gaussian decaying trend. These results led us to establish that the dynamic model on phytin conversion under phytase activation must take into account the relative diffusion between the reactant and enzyme. We believe this model may be applicable to other seeds although experimental data from more seeds are required.

## Methods

All liquid state NMR experiments were performed on a Bruker Avance 300 MHz liquid-state NMR spectrometer. All solid state NMR experiments were performed on a Varian ^UNITY^ INOVA 500 MHz solid-state NMR spectrometer and a Varian ^UNITY^ INOVA 200 MHz solid-state NMR spectrometer. For all experiments, a single 90° pulse excitation was used. For liquid-state NMR measurements, the major experimental parameters were: the 90° pulse width 10.8 μs, recycle delay 2 s, acquisition time 0.66 s and number of transients 12500.

On the solid-state NMR 500 MHz spectrometer, the major experimental parameters were: the 90° pulse width 3.5 μs, recycle delay 2 s, acquisition time 0.02 s and number of transients 4000. The sample was spun at the magic angle (MAS) with a speed from 2 kHz to 10 kHz to improve spectral resolution by removing the anisotropies from chemical shift and heteronuclear dipolar couplings between phosphorous and hydrogen. To further improve spectral resolution, a continuous heteronuclear decoupling (^31^P-^1^H) with a power of 80 kHz on ^1^H channel was also used during acquisition.

On the solid-state NMR 200 MHz spectrometer, the major experimental parameters were: the 90° pulse width 3 μs, recycle delay 1 s, acquisition time 0.03 s and number of transients 40000. The sample spinning speeds were from 2 kHz to 6 kHz. A continuous heteronuclear decoupling (^31^P-^1^H) with a power of 60 kHz on ^1^H channel was used during acquisition.

The experimental FID data were zero-filled to 4096 data points and filtered with a Lorentzian window function with a line broadening factor of 2 Hz before Fourier transform. The spectral data were then transferred to a desktop computer for further analysis.

To prevent bias in selecting sesame seeds and guarantee the representativeness and reproducibility of NMR spectra, we used tens of seeds for each germination experiment and chose the typical seeds for NMR experiments. The NMR measurement was repeated for at least two times for each spectrum. The good reproducibility was ensured. The overall errors in the germinating curves shown in Fig. 6 were less than 3%. The satisfactory agreement between experimental and theoretical data is also a good indication that the experimental data we obtained are of high quality.

## Electronic supplementary material

Supplementary information
