# Author Correction: High Resolution ^31^P NMR Spectroscopy Generates a Quantitative Evolution Profile of Phosphorous Translocation in Germinating Sesame Seed

**DOI:** 10.1038/s41598-018-31645-6

**Published:** 2018-09-04

**Authors:** Honghao Cai, Wei-Gang Chuang, Xiaohong Cui, Ren-Hao Cheng, Kuohsun Chiu, Zhong Chen, Shangwu Ding

**Affiliations:** 1 Department of Chemistry and Center for Nanoscience and Nanotechnology, National Sun Yat-sen University, 70 Lien-Hai Road, Kaohsiung, Taiwan 80424 Republic of China; 2 School of Science, Jimei University, 183 Yinjiang Road, Xiamen, China; 3 Department of Electronic Science, Fujian Provincial Key Laboratory of Plasma and Magnetic Resonance, State Key Laboratory of Physical Chemistry of Solid Surfaces, Xiamen University, Xiamen, China; 4 Department and Graduate Institute of Aquaculture, National Kaohsiung Marine University, Kaohsiung, Taiwan 811 Republic of China

Correction to: *Scientific Reports* 10.1038/s41598-017-18722-y, published online 10 January 2018

The Acknowledgements section in this Article is incomplete.

“This work was supported by the Ministry of the Republic of China to S.D. (Contract Nos NSC-103-2113-M-110-010 and MOST-104-2113-M-110-006) and K.C. (Contract No. NSC-102-2313-B-022-002), NSYSU-NKMU Joint Research Project 106-P016, the National Natural Science Foundation of China (Grant Nos. 21327001 and 11375147 to Z.C.), the Natural Science Foundation of Fujian Province (Grant No. 2016J01078 and 2017J05011 to H.C.) and the Fundamental Research Funds for the Central Universities (Grant No. 20720160125 to X.C.).”

should read:

“This work was supported by the Ministry of the Republic of China to S.D. (Contract Nos NSC-103-2113-M-110-010 and MOST-104-2113-M-110-006) and K.C. (Contract No. NSC-102-2313-B-022-002), NSYSU-NKMU Joint Research Project 106-P016, the National Natural Science Foundation of China (Grant Nos. 21327001 and 11375147 to Z.C., 11705068 to H.C.), the Natural Science Foundation of Fujian Province (Grant No. 2016J01078 and 2017J05011 to H.C.) and the Fundamental Research Funds for the Central Universities (Grant No. 20720160125 to X.C.).”

