# Supplementary material for: High Resolution 31P NMR Spectroscopy Generates a Quantitative Evolution Profile of Phosphorous Translocation in Germinating Sesame Seed
Source: Sci Rep. 2018 Jan 10;8:359. doi: 10.1038/s41598-017-18722-y (PMC5762687; doi:10.1038/s41598-017-18722-y)
Supplement: Supplementary file 1 — Supplementary information [file 41598_2017_18722_MOESM1_ESM.doc]

**Supplementary Information**

**High Resolution ^31^P NMR Spectroscopy Generates a Quantitative Evolution Profile of Phosphorous Translocation in Germinating Sesame Seed**

**Honghao Cai, Wei-Gang Chuang, Xiaohong Cui, Ren-Hao Cheng, Kuohsun Chiu*, Zhong Chen, Shangwu Ding***


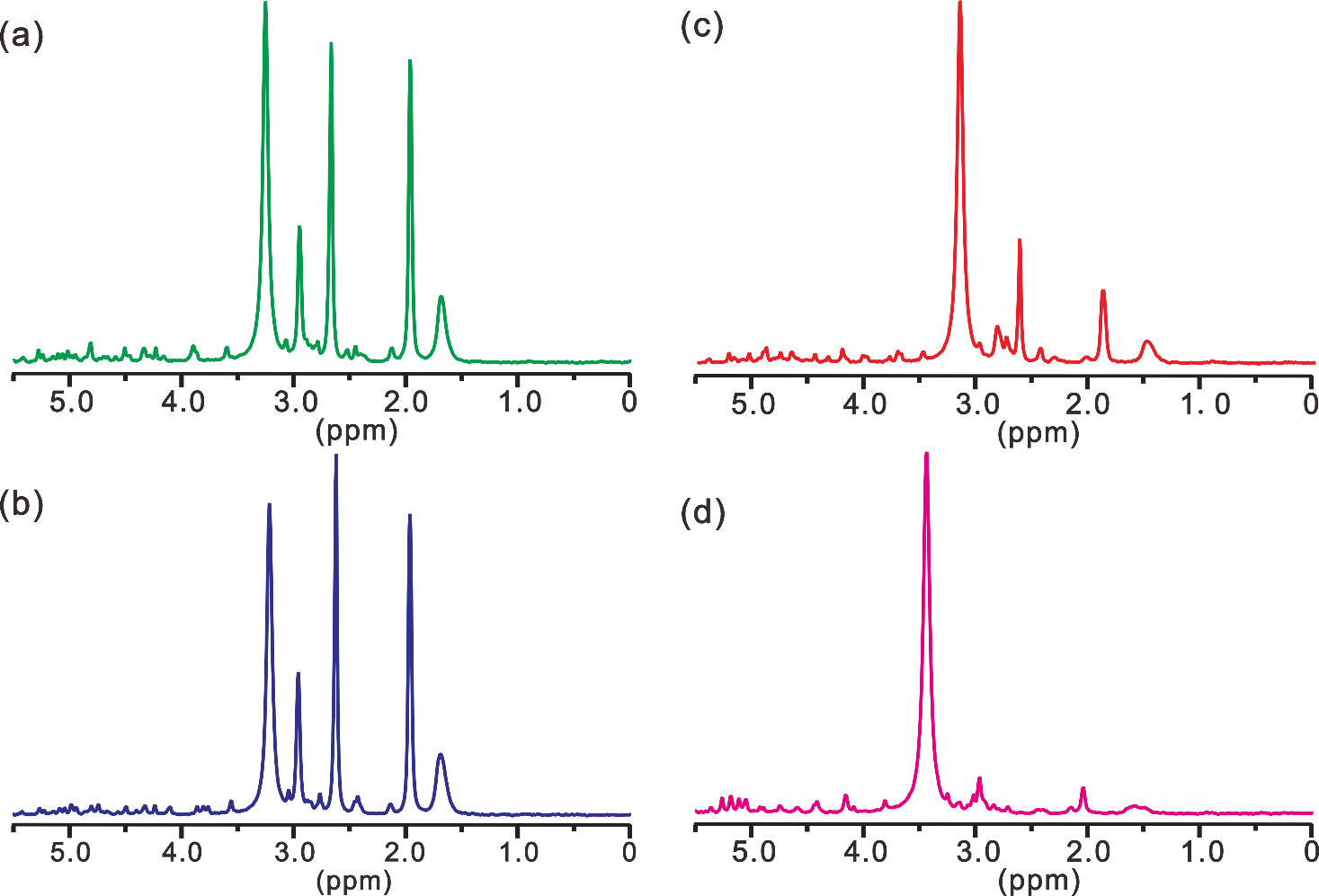


Fig. S1 The ^31^P spectra of sesame seed germinating in dark at 78 hours (A), 84 hours (B), 90 hours (C), 96 hours (D).


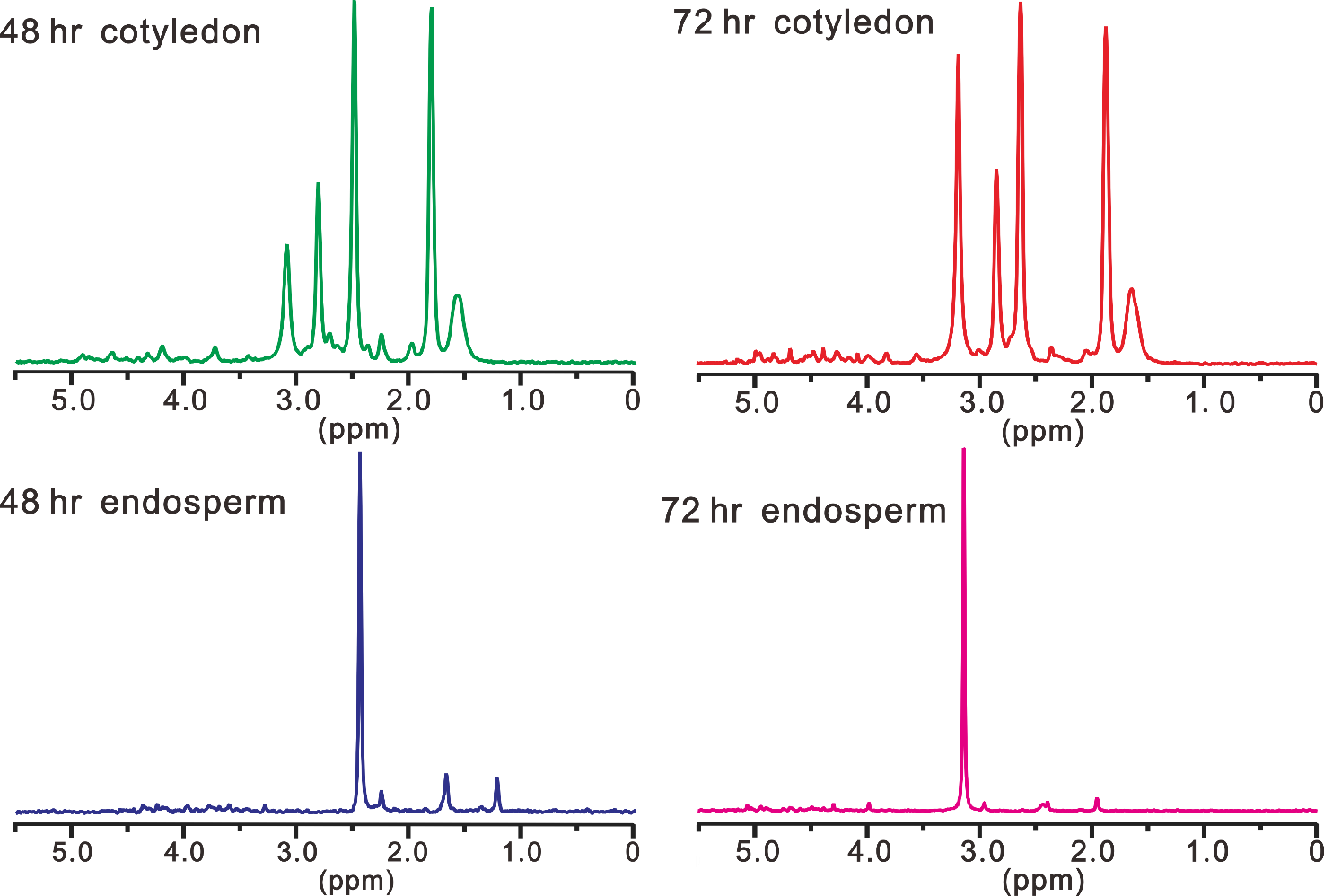


Fig. S2 The ^31^P spectra of sesame seed extracts of cotyledon and endosperm at 48 and 72 hours after germination (in dark) was initiated.


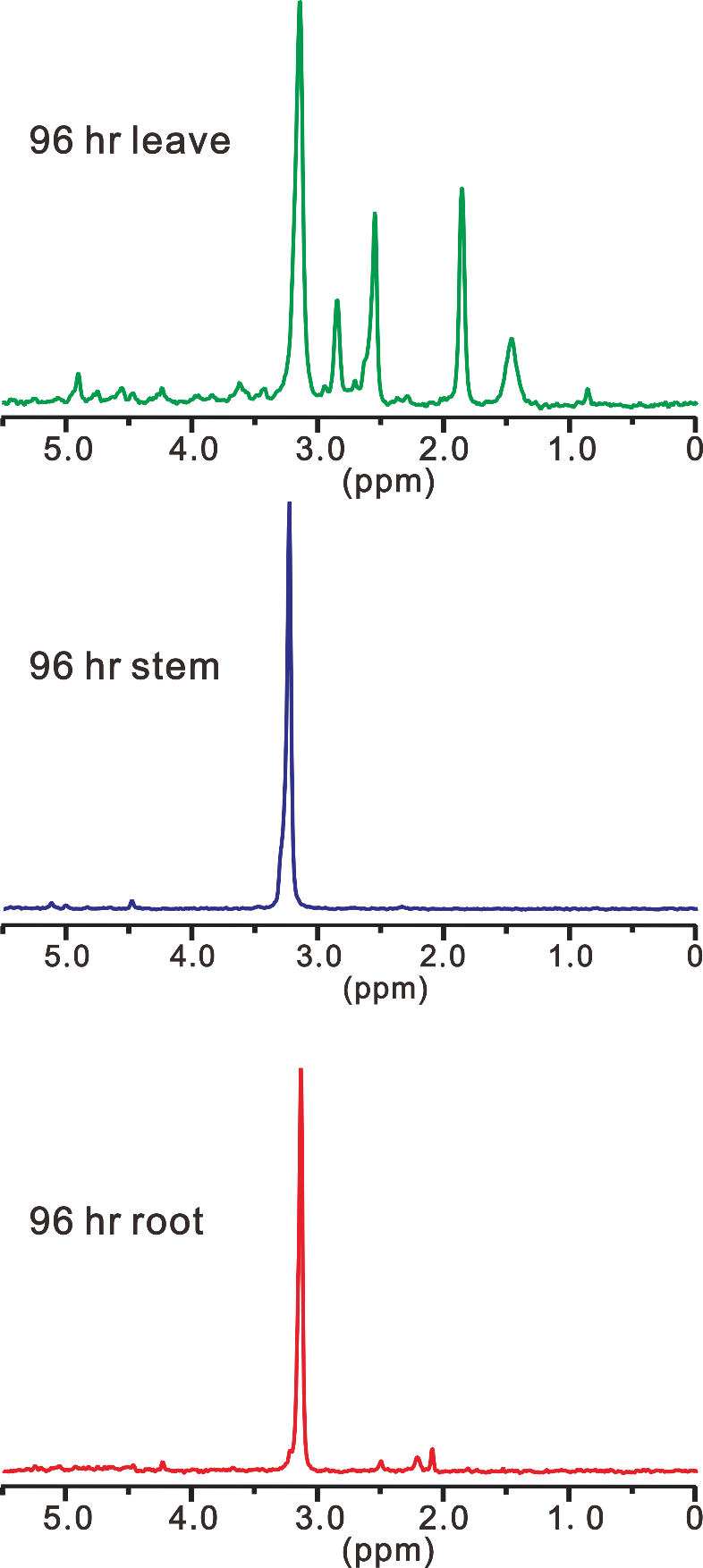


Fig. S3 The ^31^P spectra of sesame seed extracts of leave, stem and root at 96 hours after germination (in dark) was initiated.
